# Molecular subtyping of adrenocortical carcinoma reveals distinct subtypes with prognostic and therapeutic implications

**DOI:** 10.1530/ERC-25-0342

**Published:** 2026-08-14

**Authors:** Luming Wu, Tingwei Su, Cui Zhang, Lei Jiang, Jing Xie, Weiwei Zhou, Yiran Jiang, Xu Zhong, Weiqing Wang

**Affiliations:** ^1^ Department of Endocrine and Metabolic Diseases, Shanghai Institute of Endocrine and Metabolic Diseases, Ruijin Hospital, Shanghai Jiao Tong University School of Medicine, Shanghai, China; ^2^ National Clinical Research Center for Metabolic Diseases (Shanghai), Key Laboratory for Endocrine and Metabolic Diseases of the National Health Commission of the PR China, National Research Center for Translational Medicine, Shanghai Key Laboratory for Endocrine Tumor, State Key Laboratory of Medical Genomics, Ruijin Hospital, Shanghai Jiao Tong University School of Medicine, Shanghai, China; ^3^ Department of Pathology, Ruijin Hospital, Shanghai Jiao Tong University School of Medicine, Shanghai, China

**Keywords:** adrenal cortex, carcinoma, molecular genetics

## Abstract

Adrenocortical carcinoma (ACC) is a rare but aggressive malignancy with poor survival and limited treatment options. To comprehensively characterize its molecular landscape and identify clinically relevant subtypes, we performed an integrated genomic analysis – including whole-exome sequencing, RNA sequencing, and copy number variation profiling – on 61 Chinese patients with ACC. We identified recurrent mutations in TP53 (25%), CTNNB1 (15%), ZNRF3 (10%), and MEN1 (8%). Unsupervised clustering of transcriptomic data revealed four distinct molecular subtypes: cortisol-driven (CD, 14%), immune-suppressed (IS, 40%), cell cycle-altered (CCA, 22%), and immunomodulatory (IM, 24%). The CD subtype exhibited steroidogenic pathway activation; the IS subtype showed T cell receptor downregulation and the worst disease-free survival; the CCA subtype was marked by chromosomal instability and cell cycle gene overexpression; and the IM subtype displayed enriched immune signaling and favorable outcomes. Copy number analysis further uncovered focal amplifications (e.g. TERT, CDK4) and HLA-II deletions. This study establishes a novel molecular classification of ACC, providing a framework for subtype-specific therapeutic strategies, such as CDK4/6 inhibition for CCA and immunotherapy for IM tumors, while highlighting the clinical challenges of immune-cold IS tumors.

## Introduction

Adrenocortical carcinoma (ACC) is an uncommon but highly aggressive tumor arising from the adrenal cortex, with an estimated incidence of 0.5–2 cases per million individuals annually (1, 2). It accounts for approximately 0.4–4% of all adrenal tumors (3). Although surgical resection offers a potential cure in patients with localized disease, up to 40% of patients present with metastatic involvement at diagnosis (4). For advanced ACC, mitotane – either alone or in combination with etoposide, doxorubicin, and cisplatin (EDP-M) – remains the standard systemic therapy (5, 6). However, the long-term prognosis remains poor, with a five-year survival rate below 20% (7).

Immune checkpoint inhibitors (ICIs), including anti-PD-1 and anti-PD-L1 therapies, have demonstrated limited clinical benefit in unselected ACC populations (8, 9). To date, predictive biomarkers that reliably identify responders to immunotherapy are lacking. Furthermore, the heterogeneity of ACC and the scarcity of actionable genetic alterations have hindered the development of targeted therapies (10), in contrast to recent advances in precision oncology for more common malignancies.

Previous genomic studies, including those conducted by The Cancer Genome Atlas (TCGA) and other international consortia (11, 12), have identified recurrent mutations in TP53, CTNNB1, and ZNRF3, as well as distinct molecular subtypes with prognostic relevance. Notably, activating mutations in CTNNB1 – typically located in exon 3 – are present in both benign and malignant adrenal neoplasms (13). In addition, candidate oncogenic drivers such as TERT and MEN1 have been implicated in ACC progression. Despite these insights, the clinical impact of molecular classification remains limited, and practical subtype-specific treatment strategies are still lacking.

To address these challenges, we performed an integrative genomic and transcriptomic analysis of a large cohort of Chinese patients with ACC. Using whole-exome sequencing (WES), RNA sequencing, and copy number profiling, we aimed to define the molecular landscape of ACC, identify novel biologically relevant subtypes, and explore their associations with clinical outcomes. This work provides new insights into the pathogenesis of ACC and may facilitate future precision-based treatment approaches.

## Materials and methods

### Patient cohort

A total of 61 patients diagnosed with adrenocortical carcinoma between January 2023 and December 2024 at the Shanghai Clinical Center for Endocrine and Metabolic Diseases, Ruijin Hospital, were prospectively enrolled. All tumors were histologically confirmed and classified using the Weiss scoring system (score ≥ 3). Clinical staging followed the ENSAT TNM classification. None of the patients received prior oncological treatment. Tumor and matched blood samples were collected, and five-year follow-up data were recorded. The study protocol was approved by the Ethics Committee of Ruijin Hospital, Shanghai Jiao Tong University School of Medicine. Consent has been obtained from each patient or subject after full explanation of the purpose and nature of all procedures used.

### Sample processing and nucleic acid extraction

Formalin-fixed paraffin-embedded (FFPE) tumor tissues and matched peripheral blood samples were used for DNA and RNA extraction. The QIAGEN AllPrep DNA/RNA FFPE Kit was employed to isolate genomic DNA and total RNA from each sample. RNA integrity was evaluated using the Agilent 2100 Bioanalyzer, and only samples with adequate quality and at least 20% tumor cell content were included.

### DNA sequencing, genomic mutation detection, and somatic copy number alteration calling

WES was performed using the Illumina HiSeq X Ten platform with the Agilent SureSelect Human All Exon v5 (50 M) capture kit, generating 150-bp paired-end reads. Sequencing depth averaged 230× in tumor samples and 135× in matched normal tissues. Adapter trimming and quality filtering were conducted, and reads were aligned to the human reference genome (hg19) using BWA. Duplicates were marked with Picard, and local realignment around indels was performed with GATK (14). Somatic single nucleotide variants and indels were identified using MuTect and Strelka (15, 16), respectively. Artifacts and residual germline variants were filtered using an in-house false-positive database. High-confidence variants were annotated with Oncotator (17). Somatic copy number alterations (SCNAs) were detected using GATK4 Alpha (formerly ReCapSeg), and focal events were identified using the GISTIC2.0 algorithm. Tumor purity was estimated with the ABSOLUTE algorithm.

### RNA sequencing and differential expression analysis

Total RNA was extracted from formalin-fixed paraffin-embedded (FFPE) tissue specimens using a column-based protocol optimized for FFPE samples. RNA purity and concentration were assessed using the NanoDrop One spectrophotometer (Thermo Fisher Scientific, USA) and Qubit® 3.0 Fluorometer (Life Technologies, USA). RNA integrity was evaluated using the Agilent 2100 Bioanalyzer (Agilent Technologies, USA), and only samples passing minimum quality thresholds (RIN >2.0 or DV200 > 30%) were used for library construction.

Strand-specific RNA-seq libraries were generated using a dUTP-based directional protocol. Briefly, polyadenylated RNA was reverse transcribed into first-strand cDNA, followed by second-strand synthesis with dUTP instead of dTTP. After end-repair, A-tailing, and ligation of sequencing adapters, the second-strand cDNA was selectively degraded using USER enzyme treatment. The resulting single-stranded cDNA was PCR-amplified to generate indexed libraries. Library size distribution was assessed using the Agilent High Sensitivity DNA Kit on a Bioanalyzer 2100 system, and quantification was performed with the Qubit dsDNA HS Assay.

Paired-end sequencing (2 × 150 bp) was performed on the Illumina NovaSeq 6000 platform using standard protocols. Cluster generation was carried out on the cBot system, and sequencing was controlled via Illumina’s data collection software. For each sample, a minimum of 10 Gb of raw data was generated, and quality control ensured that >90% of bases achieved a Phred score of Q30 or higher. Reads were mapped to the UCSC hg19 reference genome using HISAT and Bowtie2 (18, 19). Transcript abundance was estimated using RSEM (20). Differentially expressed genes (DEGs) were identified using DEGSeq and Wilcoxon tests. DEGs were defined by a fold change ≥1.5, *q*-value < 0.1, and Wilcoxon *P*-value < 0.05.

### Clustering and subtype classification

Unsupervised k-means clustering and consensus clustering (1,000 iterations, 0.8 resampling) were applied using the ‘kmeans’ and ‘ConsensusClusterPlus’ packages in R (21). The optimal number of clusters was determined based on changes in the cumulative distribution function (CDF) and the area under the CDF curve. Genes used for clustering were selected based on expression variability from the top 5–30% standard deviation range.

### Functional enrichment analysis

Gene ontology (GO) enrichment analysis was conducted using DAVID to identify overrepresented biological processes, molecular functions, and cellular components among mutated genes. Fisher’s exact test was used to assess enrichment significance. KEGG pathway analysis was also performed using DAVID to identify functional categories and biological pathways. Pathways with *q*-values < 0.1 were considered significantly enriched.

### Statistical analysis

Kaplan–Meier survival analysis was used to estimate overall survival (OS) and disease-free survival (DFS). Differences in survival curves were compared using the log-rank test. All statistical analyses were conducted using SPSS version 25.0 (IBM Corp., Armonk, NY, USA). A two-sided *P*-value <0.05 was considered statistically significant.

## Results

### Patients’ characteristics and overview of genetic landscape

Clinical characteristics of 61 patients are summarized in Table 1. Of the participants recruited for the study, 20 were males, and 41 were females. The median age of the patients was 42 years. The median size of the adrenal mass was 7.65 cm according to the adrenal CT scan. All the masses were histologically confirmed as ACCs after operation. ACC tumor samples were obtained from patients harboring stage I (7/61 = 11.5%), stage II (35/61 = 57.4%), stage III (10/61 = 16.4%), and stage IV (6/64 = 4.9%) disease. Among the 61 included patients, WES and RNA-Seq were performed on 61 and 50 tumor samples, respectively.

**Table 1:** Overview of clinical characteristics of ACCs.

Variables	ACCs
Participants, *n*	61
Age, y	42 (31, 52)
Male, *n* (%)	20 (32.8%)
Tumor ENS@T stage, *n* (%)	
I	7 (11.5%)
II	35 (57.4%)
III	10 (16.4%)
IV	6 (9.8%)
NA	3 (4.9%)
Tumor size, mm	7.65 (6.20, 10.90)
Preoperative hormone secretion, *n*	
Yes	20
No	13
NA	28

The WES of DNA from 61 patients and matched normal pairs displayed an average sequencing depth of 230× for tumors with 98.58% of bases >10×, and 135× for normal samples, with 99.20% of bases >10× (Supplementary Table 1 (see section on Supplementary materials given at the end of the article)). We identified a total of 8,626 somatic mutations in the coding regions among the 61 samples (Supplementary Table 2), with a median of 89 non-silent mutations per tumor. The most common substitution in ACC was the C>T or G>A transition, followed by the C>A or G>T transition, which was consistent across most cancer types (Supplementary Fig. 1 and Supplementary Table 3).

### Analysis of tumor-specific mutated genes

**Figure 1:**
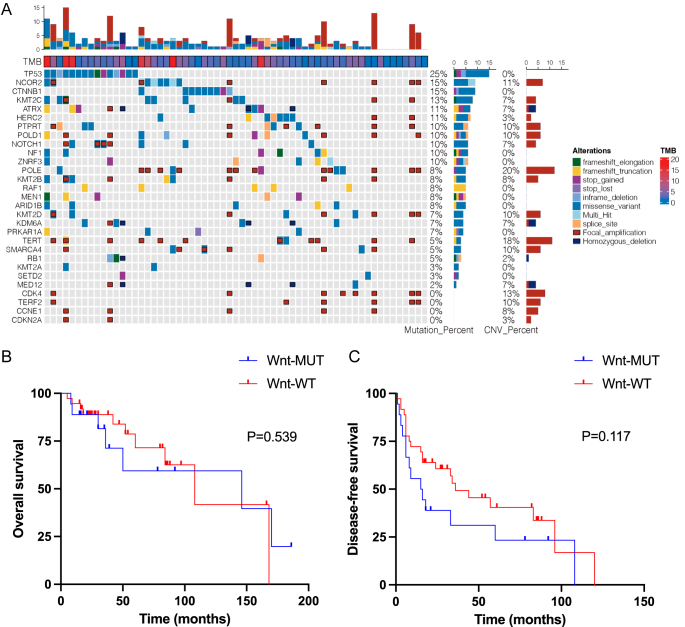
Mutational landscape of adrenocortical carcinoma (ACC). (A) Distribution of somatic mutations in key driver genes in 61 ACC tumors, identified by whole-exome sequencing (WES). The most frequent mutations include TP53 (25%), CTNNB1 (15%), ZNRF3 (10%), and MEN1 (8%). (B and C) Overall survival (OS) and disease-free survival (DFS) in our cohort based on Wnt-related mutations. A full color version of this figure is available at https://doi.org/10.1530/ERC-25-0342.

We used MuTect2 to comprehensively identify the significantly mutated genes in ACC. The ACC genomes carried a median of 72 single nucleotide variants (SNVs), 17 small insertions and deletions (InDels), and 2.665 mutations per megabase (Fig. 1A, Supplementary Table 4). Three genes were identified as recurrently altered in at least 15% of sequenced subjects, the most frequent being TP53 (25%), NCOR2 (15%), and CTNNB1 (15%). Mutations of TP53 and CTNNB1 in ACC are well recognized. As expected, the most frequent alterations were missense mutations at CTNNB1, in a hotspot region encoding serine in exon 3, resulting in an amino acid substitution (p.S45P, p.S45F, p.T41A, p.I35N, p.D32N, and p.Q26X). We also identified a deletion leading to alterations of exon 3 (p.44_45del). Six tumors harbored inactivating mutations in ZNRF3, consistent with prior studies implicating ZNRF3 in ACC. In our cohort, one patient was clinically and genetically diagnosed as MEN1 with hyperparathyroidism, ACC, and suspected pancreatic tumors. Genetic testing identified a germline heterozygous mutation in the MEN1 gene, c.1453dupT. Moreover, five (8%) cases harbored inactivating somatic mutations in MEN1. There was no significant difference in age, gender, and tumor size between patients with Wnt-related mutations and Wnt WT patients in the cohort. Fourteen (87.5%) patients were identified with recurrence in the Wnt-related mutation group, compared with 20 (44.4%) patients in the Wnt WT group (*P* = 0.003); 7 (43.8%) patients with Wnt-related mutations died, compared with 11 (24.4%) patients in the Wnt WT group (*P* = 0.252) (Table 2). Furthermore, we compared overall survival (OS) (Fig. 1B) and disease-free survival (DFS) (Fig. 1C) in our cohort based on Wnt-related mutations. Patients with Wnt-related mutations showed a reduced median DFS (15.5 vs 36 months), with a 5-year survival of 70%.

**Table 2:** Wnt mutations in ACCs.

Variables	Wnt-related mutation (*n* = 16)	Wnt WT (*n* = 45)	*P*-value
Age, y	43.0 (30.5, 52.0)	39.0 (34.5, 54.3)	0.876
Male, *n* (%)	7 (43.8%)	13 (28.9%)	0.277
Tumor diameter, cm	8.00 (6.45, 10.90)	7.50 (5.40, 11.70)	0.56
Recurrence, *n* (%)	14 (87.5%)	20 (44.4%)	0.003
Death, *n* (%)	7 (43.8%)	11 (24.4%)	0.252
Systemic therapies, *n* (%)			
Platinum-based chemotherapy, *n* (%)	6 (37.5%)	10 (22.2%)	0.233
Mitotane, *n* (%)	15 (93.8%)	33 (73.3%)	0.087
Immunotherapy, *n* (%)	9 (56.3%)	13 (28.9%)	0.05

### Population-based differences in ACC driver gene alterations

To investigate population-specific genomic features of ACC, we compared the overall alteration frequencies (including somatic mutations, homozygous deletions, and high-level amplifications) of key driver genes in our Chinese cohort (Ruijin, *n* = 61) with those reported in the ENSAT (*n* = 122) and TCGA (*n* = 90) cohorts (Table 3).

**Table 3:** Distribution of driver gene alterations in ACC cohorts.

Gene	ENSAT (*n* = 122)	TCGA (*n* = 90)	RUIJIN (*n* = 61)
ZNRF3	26 (21%)	17 (19%)	6 (10%)
CTNNB1	20 (16%)	14 (16%)	9 (15%)
TP53	20 (16%)	19 (21%)	15 (25%)
CDKN2A	13 (11%)	14 (16%)	2 (3%)
RB1	9 (7%)	6 (7%)	4 (7%)
MEN1	9 (7%)	6 (7%)	5 (8%)
DAXX	7 (6%)	0	0
MED12	6 (5%)	0	5 (8%)
PRKAR1A	0	10 (11%)	4 (7%)
RPL22	0	7 (8%)	0
NF1	0	7 (8%)	6 (10%)

TP53 was altered in 25% of our patients, which is higher than the frequencies observed in ENSAT (16%) and TCGA (21%). CTNNB1 alterations were present in 15% of Chinese cases, comparable to both reference cohorts (16% in each). Notably, ZNRF3 alterations were less frequent in our cohort (10%) than in ENSAT (21%) and TCGA (19%), suggesting potential population-specific differences in Wnt pathway dysregulation. In contrast, NF1 (10%) and PRKAR1A (7%) showed alteration rates similar to those reported in TCGA (8 and 11%, respectively). CDKN2A alterations were markedly lower in our patients (3%) compared with ENSAT (11%) and TCGA (16%).

Our findings reveal population-specific differences in oncogenic mechanisms, emphasizing the importance of ethnicity-tailored molecular profiling for precision treatment in ACC.

### Copy number variation in ACCs

**Figure 2:**
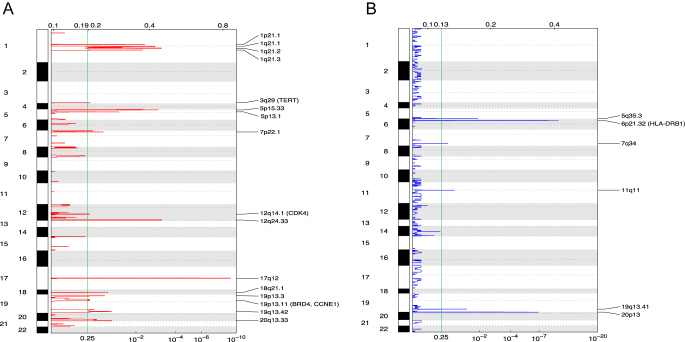
Somatic copy number alterations (CNAs) in ACC. (A) Heatmap of significant somatic copy number variations (CNVs) detected in 61 ACC tumor samples using GISTIC2.0. Recurrent amplifications were observed at loci TERT (5p15.33), CDK4 (12q14.1), and CCNE1 (19q12). (B) Chromosomal regions with significant losses (HLA-DQA1, HLA-DRB1, HLA-DRB5 on 6p21.32 and 20p13). A full color version of this figure is available at https://doi.org/10.1530/ERC-25-0342.

We next assessed somatic copy number variations (CNVs) in 61 FFPE samples. Statistically significant focal amplified regions and deleted regions were detected using GISTIC2.0. The most significant DNA copy number changes in ACC included gains of 17q12, 12q24.33, 5q15.33, 1p21.1, and 1q21.2 (Fig. 2). We identified recurrent focal amplifications of TERT (5p15.33), CDK4 (12q14.1), and CCNE1 (19q12) (Supplementary Table 5). Although not statistically significant, focal peak gains or amplifications were found in 6 patients (10%) in the 16q22 region, including TERF2, which is located nearby. TERT and TERF2, two telomere-maintenance-related genes, were focally amplified in 18 and 10% of cases, respectively. We also specifically examined the ZNRF3 locus (22q11.23) for copy number alterations; however, no homozygous or heterozygous deletions were detected in any of the 61 tumors, and no focal amplifications involving ZNRF3 were observed. The most strikingly deleted regions were losses of 6p21.32 and 20p13, in which five HLA-related genes (HLA-DQA1, HLA-DQB1, HLA-DRB1, HLA-DRB5, and HLA-DRB6) were involved (Supplementary Table 6). These results suggest that HLA-related genes may be important in ACCs.

### Transcriptomic profiles of the ACC cohort

**Figure 3:**
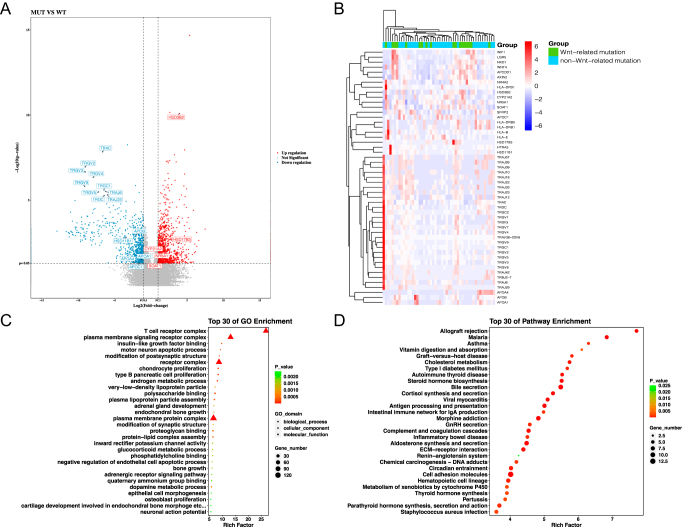
Transcriptomic profiling and differentially expressed genes (DEGs). (A) Volcano plot showing differentially expressed genes (DEGs) between Wnt-related mutant and wild-type ACC tumors. (B) Heatmap of Wnt-related mutant and wild-type ACC tumor tissues with the variable DEGs. Every row represents a gene, and each column represents a sample. (C) Gene ontology (GO) enrichment analysis of DEGs, showing significant downregulation of T cell receptor genes in Wnt-related mutant tumors. (D) KEGG pathway enrichment analysis indicating the activation of steroid hormone biosynthesis pathways in Wnt-mutant tumors. A full color version of this figure is available at https://doi.org/10.1530/ERC-25-0342.

We analyzed RNA-seq data and obtained differentially expressed genes (DEGs) between Wnt-related mutant and wild-type ACCs (Fig. 3A, Supplementary Tables 7 and 8). Of note, we did not observe a significantly expressed change in CTNNB1, which was further supported by qPCR quantification. The DEGs (HSD3B2, CYP21A2, HSD17B3, NR5A1, HSD11B1, and SOAT1) involved in steroidogenesis were significantly different expressed in Wnt-related mutant ACCs (Fig. 3B). GO enrichment and KEGG pathway analysis were then applied to assess the enriched signaling pathways of these DEGs. The GO analysis showed that these mutated genes were enriched in ‘T cell receptor complex’. TRAC and TRGV2/3/4/9 involved in the term were significantly downregulated in Wnt-related mutant ACCs (Fig. 3C, Supplementary Table 9). These genes may play critical roles in T cell development, cancer immunosurveillance, and autoimmunity according to previous publications. The KEGG analysis demonstrated that the DEGs were enriched in the cholesterol metabolism pathway and steroid hormone biosynthesis pathway (Fig. 3D, Supplementary Table 10). Our results indicate that Wnt-related mutation triggers steroid hormone biosynthesis and T cell-mediated antitumor immunity.

### ACC subtypes based on multi-omics data

We performed consensus clustering by resampling (1,000 iterations) randomly selected tumor profiles (Supplementary Fig. 2A). According to the ‘elbow’ point in the relative change in area under the consensus distribution function plot (Supplementary Fig. 2B), we chose four as the number of subtypes. Based on these findings, we classified 50 tumors into 4 separate subtypes using unsupervised k-means clustering. These four subtypes consisted of the following: i) a cortisol driven (CD) subtype (14%) characterized by upregulation of cortisol synthesis and secretion; ii) an immune-suppressed (IS) (comprising 40% of tumors) subtype characterized by downregulation of immune response genes; iii) a cell cycle alteration (CCA) subtype (22%) characterized by upregulation of cell cycle, and downregulation of ATP-dependent chromatin remodeling; iv) immunomodulatory (IM) subtype (24%) with high immune cell signaling and cytokine signaling gene expression (Table 4). Several candidate gene targets were identified within each subtype, indicating the potential application of future subtype-specific precision treatments.

**Table 4:** Highlights of genomic, clinical, and potential treatment strategies for ACC subtypes.

Subtype	CD	IS	CCA	IM
Clinical	Good prognosis (5-year PFS, 43%)	Poor prognosis (5-year PFS, 15%)	Poor prognosis (5-year PFS, 18%)	Good prognosis (5-year PFS, 75%)
Mutation	TP53 (29%) Wnt-related mutations (29%)	TP53 (35%) Wnt-related mutations (45%)	TP53 (9%) Wnt-related mutations (18%)	TP53 (0%) Wnt-related mutations (33%)
Copy number	Focal amplifications of TERT and TERF2 (14%)	Focal amplifications of TERT and TERF2 (20%)	Focal amplifications of TERT and TERF2 (45%)	Focal amplifications of TERT and TERF2 (8%)
KEGG pathway analysis	Ferroptosis upregulated	Type 1 diabetes mellitus downregulated	p53 signaling pathway upregulated	Neutrophil extracellular trap formation downregulated
	Cortisol synthesis and secretion upregulated	Allograft rejection downregulated	ATP-dependent chromatin remodeling downregulated	Systemic lupus erythematosus downregulated
Treatment	Targeted ferroptosis inducers, GR antagonists	Escalated chemotherapy, intensive monitoring	CDK 4/6 inhibitors, cell cycle inhibitors	Immune checkpoint blockade

**Figure 4:**
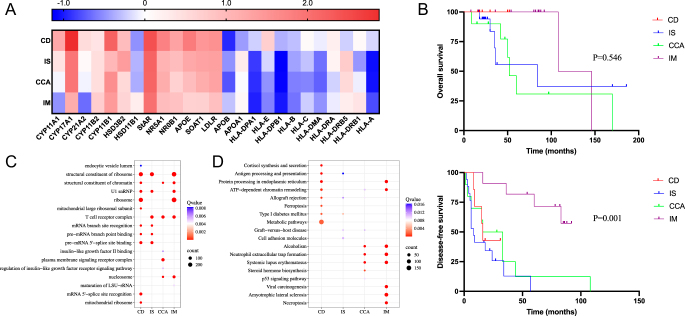
Molecular features and prognosis of ACC subtypes. (A) Summary of key molecular features across the four ACC subtypes: CD, IS, CCA, and IM, based on gene expression. (B) Comparison of recurrence rates and overall survival (OS) across the four subtypes, demonstrating that IS tumors have the highest recurrence rates, while IM tumors have the most favorable prognosis. (C) Overview of the molecular pathways activated in each subtype: CD subtype shows increased steroidogenesis and mitochondrial pathways, IS subtype has altered immune response pathways, and CCA subtype displays cell cycle-related changes and upregulated immune-related genes in IM subtype. A full color version of this figure is available at https://doi.org/10.1530/ERC-25-0342.

Since steroidogenesis and immune-related gene expression have been proposed as features of ACC. By comparing DEGs among the four subtypes, we found that CYP11A1, CYP17A1, and CYP11B1 expressions were upregulated in the CD subtype, while the IM subtype demonstrated a high expression of HLA-DRB1 and HLA-DRB5 (Fig. 4A).

IM subtype had significantly good DFS compared with patients in other groups (*P* = 0.001, Fig. 4B). In contrast, Kaplan–Meier survival analysis revealed no significant difference in OS among the four subtypes (Fig. 4B).

### Molecular features of ACC subtypes

The GO analysis showed that CD subtype showed these genes enriched in ‘mitochondrial ribosome’, CCA subtype was enriched in ‘insulin-like growth factor II binding’ and IM subtype was enriched in ‘T cell receptor complex’ (Fig. 4C). The KEGG analysis demonstrated that the DEGs were enriched in the cortisol synthesis and secretion and ferroptosis in CD subtype, type 1 diabetes mellitus and allograft rejection in IS subtype, p53 signaling pathway and ATP-dependent chromatin remodeling in CCA subtype, neutrophil extracellular trap formation, and systemic lupus erythematosus in IM subtype (Fig. 4D). Our results indicate that steroid hormone biosynthesis and T cell-mediated antitumor immunity were important for ACC.

The CD subtype harbored driver genomic events, such as TP53 (29% in CD, 35% in IS, 9% in CCA, and 0% in IM) and Wnt-related mutations (29% in CD, 45% in IS, 18% in CCA, and 33% in IM) at a frequency between IS and the other two subtypes. Gene expression profiling revealed that the CD subtype displayed characteristics of cortisol synthesis and secretion. To further understand the alterations in this subtype, the steroidogenesis enzymes that play a crucial role were investigated. The results showed that this subtype exhibited a higher expression of StAR, CYP11A1, CYP21A2, HSD3B2, CYP17A1, CYP11B1, NR5A1, and NR0B1; these data suggest that the identification of the CD subtype may be beneficial for targeting cortisol synthesis inhibitors as a potential treatment strategy.

IS subtype indicates a group of ACCs with a worse prognosis and lacks immune activation; hence, it is not likely to benefit from immune checkpoint inhibitors. Seventeen out of 20 (85%) patients who experienced recurrence received subsequent treatment. Among the 20 patients, 7 carried TP53 mutations, and 9 had Wnt-related mutations. Gene expression profiling revealed that the IS subtype exhibited downregulation of the T cell receptor complex, suggesting suboptimal response to immune checkpoint inhibitors and necessitating alternative antitumor therapies such as first-line chemotherapy and more intensive tumor surveillance.

CCA subtype was characterized by recurrent copy number alterations (CNAs) and activation of the cell cycle pathway. In 9 out of 11 (82%) patients, the condition recurred within 5 years after surgery. Expression analyses also showed increased mRNA expression levels for CCND1 and E2F1 in CCA tumors. These data suggested potential sensitivity of CCA tumors to CDK 4/6 inhibitors or other cell cycle inhibitors.

Despite the relatively favorable outcome of the immunomodulatory (IM) subtype, 25% of patients in this group experienced recurrence and/or metastases within 5 years after surgery. This subtype was characterized by elevated immune cell signaling, as observed in the gene expression data. Notably, TRBV and TRAV gene families were significantly overexpressed in the IM subtype, suggesting an active and diverse T cell receptor (TCR) repertoire. This increased TCR diversity may reflect enhanced immune surveillance and a more effective antitumor immune response. The KEGG pathway analysis demonstrated the downregulation of neutrophil extracellular trap formation, PD-L1 expression, and PD-1 checkpoint pathway in cancer between IM versus other subtypes, providing additional rationale for the use of an immune checkpoint blockade as a therapeutic approach.

## Discussion

Consistent with prior studies such as those from The Cancer Genome Atlas (TCGA) and the ENSAT network (11, 12), our cohort exhibited frequent alterations in canonical driver genes, including TP53 (25%), CTNNB1 (15%), and ZNRF3 (10%). These findings support the central role of p53 and Wnt/β-catenin pathway dysregulation in ACC tumorigenesis. Mutations in CTNNB1 were clustered in exon 3, consistent with gain-of-function events known to drive nuclear β-catenin accumulation (22). Notably, our data also identified recurrent mutations in NCOR2 (15%), a transcriptional corepressor not previously emphasized in ACC, raising the possibility of alternative epigenetic regulation contributing to tumor behavior in certain subgroups (23, 24).

Our findings are largely consistent with previous international studies such as those from The Cancer Genome Atlas (TCGA) (11), while also providing new population-specific insights. In addition to confirming recurrent mutations in TP53, CTNNB1, and ZNRF3, our data reveal population-specific variation in driver gene frequencies. Compared with the ENSAT (*n* = 122) and TCGA (*n* = 91) cohorts, Chinese patients with ACC in our cohort showed a higher frequency of TP53 alterations (25 vs 16% in ENSAT and 21% in TCGA). CTNNB1 alterations were comparable across cohorts (15% in our cohort vs 16% in both reference cohorts). Notably, ZNRF3 alterations were less frequent in our patients (10%) than in ENSAT (21%) and TCGA (19%), suggesting that East Asian patients may rely less on ZNRF3 loss as a driver of Wnt pathway dysregulation. These findings highlight the necessity of population-specific genomic reference maps to improve the equity and accuracy of precision oncology in rare tumors such as ACC.

In our study, Wnt/β-catenin mutations were found to be associated with poor clinical outcomes, particularly shortened disease-free survival (DFS), with patients harboring these mutations exhibiting a median DFS of 15.5 months, compared with 36 months for those without Wnt-related mutations. This association between CTNNB1 mutations and worse survival has been previously reported by Gaujoux et al. in adrenocortical carcinoma (25). These findings suggest that Wnt/β-catenin dysregulation contributes to a more aggressive disease phenotype in ACC, likely due to its effects on tumor growth, cell cycle progression, and immune evasion (26, 27). The observed poor prognosis associated with Wnt/β-catenin mutations highlights the importance of incorporating molecular profiling into routine clinical practice for patients with ACC, especially for those in the early stages of the disease. Furthermore, Tissier et al. demonstrated that abnormal β-catenin immunohistochemistry correlates with somatic CTNNB1 mutations in ACC, offering a more accessible and cost-effective tool than comprehensive molecular profiling for routine clinical use (28). Targeting the Wnt/β-catenin pathway may offer new avenues for precision medicine in ACC, particularly for patients who exhibit Wnt-related mutations (29, 30).

The Wnt/β-catenin signaling pathway is known to regulate several key aspects of tumor progression, including cell proliferation, survival, and metastasis (31, 32, 33). In many tumors, Wnt/β-catenin activation has been linked to immune exclusion, particularly through the suppression of CD8+ T cell infiltration (34, 35). For instance, CTNNB1 mutation-mediated transcriptional activation of MMP9 suppressed infiltration and cytotoxicity of CD8(+) T cells, leading to drive the suppressive tumor immune microenvironment (TIME) and anti-PD-1 resistance (36). These findings have extended to adrenocortical carcinoma (ACC), where Wnt pathway activation has been shown to correlate with an immune-depleted tumor microenvironment, as demonstrated by Thorsson et al., who linked CTNNB1 mutations to immune suppression in ACC (37). Wnt-related mutations in our cohort were associated with distinct transcriptomic alterations, notably involving steroidogenic and immune pathways. Tumors harboring Wnt alterations exhibited upregulation of steroidogenesis-related genes, such as HSD3B2, CYP21A2, and NR5A1, while concurrently showing suppression of key T cell receptor components (TRAC, TRGV2/3/4/9). This co-occurrence of endocrine activation and immune evasion suggests that aberrant Wnt signaling may underlie an ‘immune-cold, hormone-active’ tumor phenotype, which may contribute to therapeutic resistance. This is in line with findings from Mohan et al., who reported that CIMP-high ACC tumors exhibited poor immune infiltration and pronounced cell cycle activation (38).

To further dissect the heterogeneity of ACC, we performed unsupervised consensus clustering of transcriptomic profiles, which yielded four reproducible molecular subtypes: cortisol-driven (CD), immune-suppressed (IS), cell cycle-altered (CCA), and immunomodulatory (IM). Each subtype exhibited distinct genomic alterations, transcriptomic signatures, and clinical outcomes, underscoring the potential of subtype-based stratification to guide personalized treatment strategies.

Compared with the three-subtype model described in The Cancer Genome Atlas cohort (C1A, C1B, and C2) (11), our four-subtype classification provides greater resolution, particularly in delineating the spectrum of immune activity and steroidogenic signatures. For example, our IS subtype exhibited broad downregulation of immune-related pathways along with frequent Wnt pathway mutations and deletions at HLA class II loci. This mirrors the ‘immune-excluded ACC’ phenotype, which was characterized by impaired antigen presentation and low T cell infiltration (39).

The CD subtype (14%) was characterized by upregulation of steroidogenic enzymes, such as CYP11A1, CYP17A1, and NR5A1. Patients in this group frequently harbored TP53 and Wnt-related mutations, consistent with cortisol excess and proliferative signatures. This subtype parallels the ‘steroid-high + proliferative’ phenotype previously described in CIMP-high tumors by TCGA, which was associated with early recurrence and poor overall survival. Recent findings highlight a unique vulnerability of adrenal cortex cells to ferroptosis, driven by their high metabolic activity in steroid hormone synthesis (40). Our results similarly demonstrate that the CD subtype exhibits significant upregulation of ferroptosis, which aligns with the steroidogenesis-dependent ferroptosis sensitivity observed in adrenal cortical cells. A previous study showed that patients with glucocorticoid excess had a particularly poor overall survival (41); due to the upregulation of steroidogenesis enzymes, CD tumors may benefit from inhibitors of steroid biosynthesis or glucocorticoid receptor antagonists.

The IS subtype (40%) was associated with the poorest disease-free survival, alongside downregulation of immune-related pathways and reduced expression of PD-L1 and PD-1. This immunologically ‘cold’ phenotype may contribute to the limited efficacy of immune checkpoint inhibitors observed in these tumors. Frequent co-occurrence of TP53 and Wnt-related alterations further suggests a complex interplay between genomic instability and immune escape, necessitating the development of alternative therapeutic approaches for this subgroup. Our team has initiated a single-center clinical trial (NCT06141369) investigating an mRNA neoantigen vaccine for ACC, with the goal of enhancing therapeutic efficacy and extending survival through personalized tumor-specific immunotherapy.

The CCA subtype (22%) was defined by the activation of the cell cycle pathway, recurrent CNVs, and the upregulation of CCND1 and E2F1. These features suggest potential sensitivity to CDK4/6 inhibitors or other agents targeting proliferative signaling. Interestingly, most patients in this group experienced recurrence within five years, despite no significant difference in overall survival. This supports a rationale for incorporating cell cycle-targeted therapeutics into clinical trials for ACC, especially in early-stage patients with CCA subtype tumors.

In our analysis of the IM subtype (24%), we observed a favorable prognosis and exhibited high expression of immune signaling genes, such as HLA-DRB1, HLA-DRB5, and TCR genes, particularly the TRBV and TRAV families. These genes, which are part of the T cell receptor (TCR) variable regions (42), were significantly overrepresented in the IM subtype compared with the other subtypes, suggesting that T cells within this subgroup are highly active and may contribute to the robust immune response observed in these tumors (43). KEGG pathway analysis identified enrichment in neutrophil extracellular trap (NET) formation and systemic autoimmune pathways, suggesting a more ‘immune-active’ microenvironment (44). The diversity of the TCR of tumor-infiltrating T cells at baseline is prognostic in various cancers, whereas the TCR clonality of T cells infiltrating metastatic melanoma pre-treatment is predictive of activity and efficacy of PD-1 blockade immunotherapy (45, 46). Patients in IM subtype may represent ideal candidates for immune checkpoint blockade, and their favorable survival outcomes further support the biological relevance of immune infiltration in ACC. Nevertheless, approximately one-quarter of patients in this group still experienced recurrence, underscoring the need for improved surveillance.

Of particular interest is the identification of somatic copy number alterations involving TERT, CDK4, and TERF2, genes associated with telomere maintenance and proliferation. In contrast, we observed focal deletions in HLA class II genes (HLA-DQA1, HLA-DRB1, HLA-DRB5), particularly in IS tumors, which may mechanistically explain immune evasion through loss of antigen presentation. These CNVs are consistent with prior pan-genomic characterizations of ACC and further emphasize the convergence of endocrine, proliferative, and immune pathways in disease progression.

Our study has several limitations. Although the cohort is one of the largest in East Asia, validation in larger and more ethnically diverse populations is needed. Functional assays were beyond the scope of this study, and future work should aim to test subtype-specific vulnerabilities in preclinical models. Furthermore, prospective trials are required to evaluate the clinical utility of molecular classification in guiding treatment decisions.

In conclusion, our study defines four transcriptomic subtypes of ACC with distinct genomic and biological features, providing a framework for future personalized therapeutic approaches. Stratification based on molecular features – particularly immune-related and steroidogenic signatures – may help identify patients most likely to benefit from immunotherapy, endocrine therapy, or targeted agents. As precision oncology efforts in rare cancers advance, integrated molecular profiling should become an essential component of ACC management.

## Supplementary materials





## Declaration of interest

The authors declare that they have no known competing financial interests or personal relationships that could have appeared to influence the work reported in this paper.

## Funding

This project received grants from the National Natural Science Foundation of China (82403817 to Dr Wu), the Shanghai Municipal Health Commission (20224Y0088 to Dr Wu and 202440098 to Dr Zhong), the Shanghai Municipal Science and Technology Commission (24QA2705200 to Dr Zhang), and the Shanghai Shenkang Hospital Development Center (SHDC2024CRI081 to Dr Wu, SHDC12025146 to Dr Zhang, and SHDC22025304 and SHDC2025CCS022 to Dr Wang).

## Author contribution statement

LW was involved in writing the original draft of the manuscript, data curation, formal analysis, investigation, and funding acquisition. TS curated the data, contributed resources, and conducted investigation, visualization, and validation. CZ designed the methodology and investigation for this study and acquired funding. LJ investigated and administered the project and contributed resources. JX and YJ designed the methodology. WZ designed the methodology and contributed software. XZ performed the procedure, validated the study, and acquired funding. WW wrote, reviewed, and edited the manuscript, administered the project, supervised and visualized the study, and was involved in funding acquisition.
