# Supplementary material for: Molecular subtyping of adrenocortical carcinoma reveals distinct subtypes with prognostic and therapeutic implications
Source: Endocr Relat Cancer. 2026 Aug 14;33(8):e250342. doi: 10.1530/ERC-25-0342 (PMC13482824; doi:10.1530/ERC-25-0342)

Supplementary Figures

Supplementary Figure 1. Somatic mutation characteristics in adrenocortical carcinoma (ACC).

Bar plot displaying the distribution of single nucleotide variant (SNV) types among the ACC cohort. C>T transitions were the most common. Boxplot illustrating the number of nonsynonymous somatic mutations per tumor, reflecting variation in somatic mutation burden across samples.

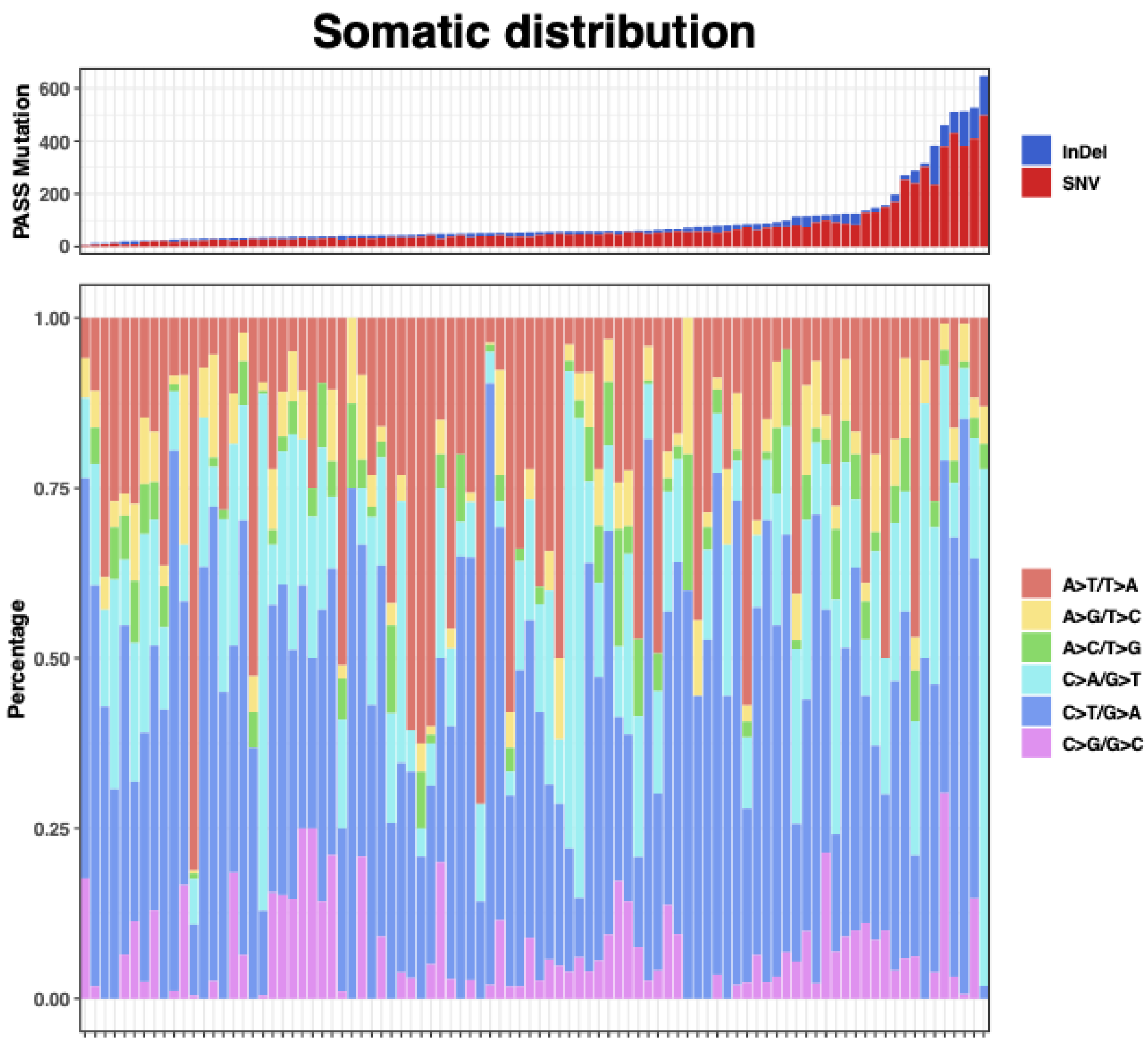

Supplementary Figure 2. Identification of transcriptomic subtypes in ACC by consensus clustering.

(A) Consensus matrix heatmap showing the results of 1,000 iterations of k-means clustering with resampling. The degree of sample co-clustering across iterations supports a four-subtype solution.

(B) Cumulative distribution function (CDF) plot of consensus indices. The inflection point in the curve suggests k = 4 as the optimal number of clusters.

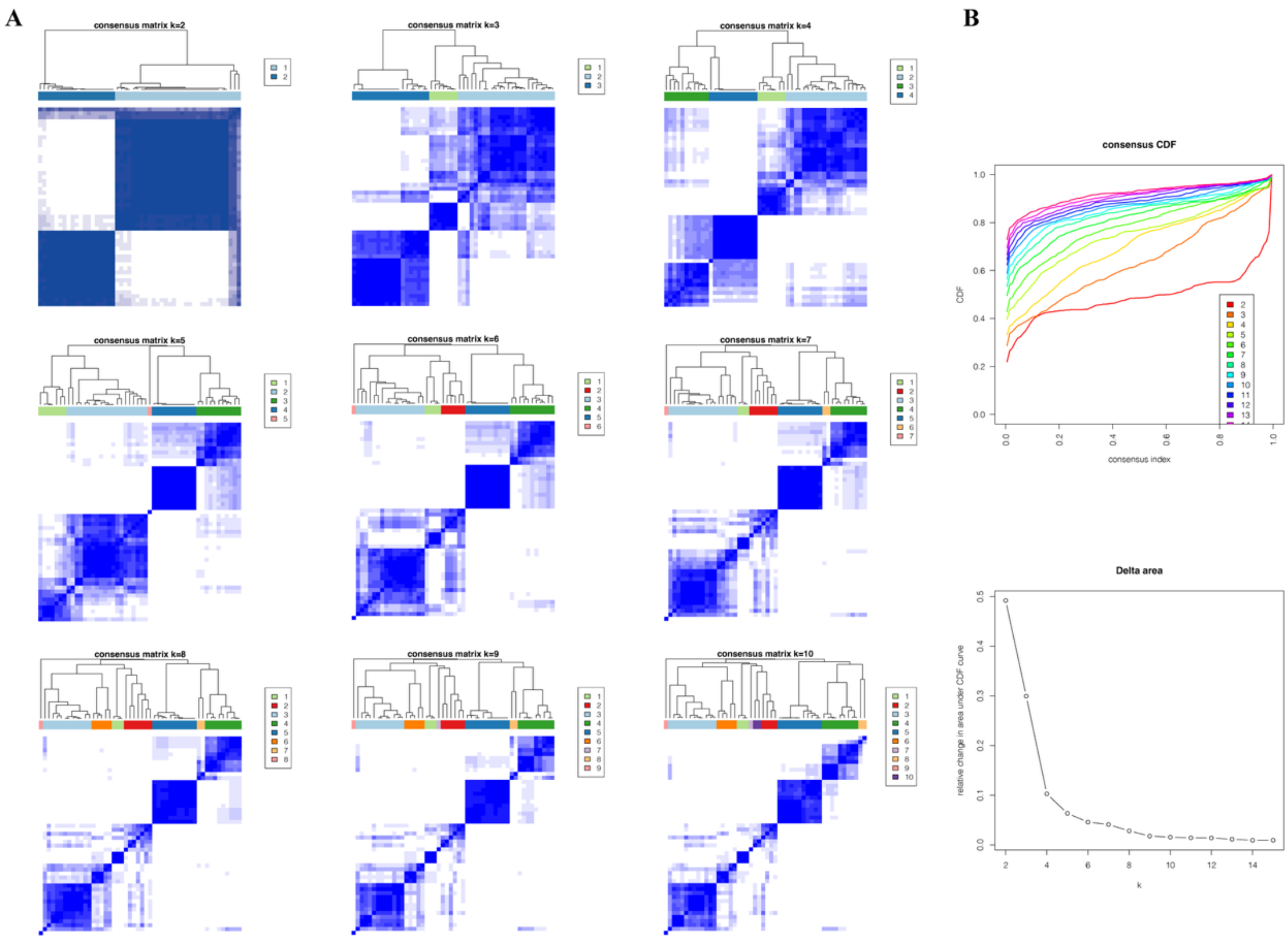

Supplement: Supplementary file 1 [file ERC-25-0342_supplementary_figures.pdf]
